# Supplementary material for: Circadian disturbances and frailty risk in older adults
Source: Nat Commun. 2023 Nov 16;14:7219. doi: 10.1038/s41467-023-42727-z (PMC10654720; doi:10.1038/s41467-023-42727-z)
Supplement: Supplementary file 1 — Supplementary Information [file 41467_2023_42727_MOESM1_ESM.pdf]

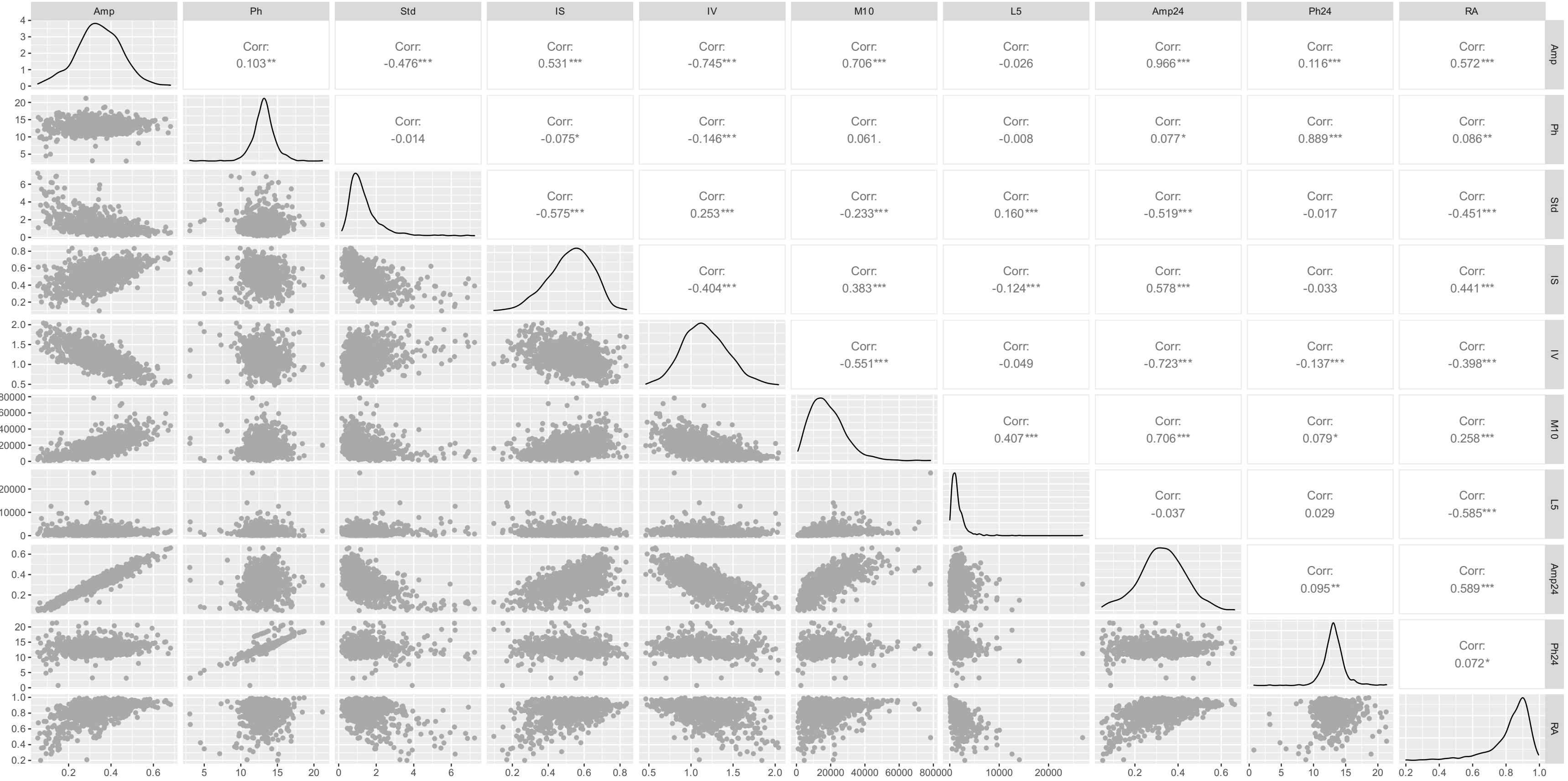

**Supplementary Fig. 1: Pairwise correlations of circadian rest-activity metrics.** Circadian rest-activity measures were derived from actigraphy recordings at analytical baseline. Scatter plots and correlation coefficient are shown separately on the corresponding symmetrical panels along the main diagonal. Baseline distributions of circadian measures are shown in the diagonal. Abbreviations: Amp, amplitude from UP-EMD; Ph, acrophase from UP-EMD; Std, variation of cycle length; IS, interdaily stability; IV, intradaily variability; M10, the average activity during the most active 10-h; L5, the average activity during the least active 5-h period; Amp24, 24-h amplitude from cosinor; Ph24, 24-h acrophase from cosinor; RA, relative amplitude.

***$p$ value < 0.001, ** $p$ value < 0.01, * $p$ value < 0.05

Source data are provided as a Source Data file.

**Supplementary Table 1. Association of primary circadian rest-activity metrics and incident frailty after controlling for demographics**

| | Amplitude | Acrophase | Variation of cycle length | IS | IV | M10 | L5 |
|---|---|---|---|---|---|---|---|
| | HR (95%CI) *p* value | HR (95%CI) *p* value | HR (95%CI) *p* value | HR (95%CI) *p* value | HR (95%CI) *p* value | HR (95%CI) *p* value | HR (95%CI) *p* value |
| Age[a] | 1.09 (1.07-1.11) <0.001 | 1.10 (1.08-1.12) <0.001 | 1.10 (1.08-1.12) <0.001 | 1.10 (1.08-1.12) <0.001 | 1.09 (1.07-1.11) <0.001 | 1.09 (1.07-1.11) <0.001 | 1.10 (1.08-1.12) <0.001 |
| Female sex | 3.54 (2.50-5.01) <0.001 | 3.12 (2.21-4.40) <0.001 | 3.52 (2.48-5.02) <0.001 | 3.40 (2.41-4.82) <0.001 | 3.43 (2.42-4.85) <0.001 | 3.52 (2.49-4.98) <0.001 | 3.26 (2.31-4.59) <0.001 |
| Education[b] | 1.06 (1.02-1.11) 0.002 | 1.05 (1.01-1.09) 0.020 | 1.05 (1.01-1.10) 0.010 | 1.06 (1.02-1.10) 0.007 | 1.06 (1.02-1.10) 0.005 | 1.05 (1.01-1.09) 0.013 | 1.04 (1.00-1.09) 0.030 |
| Amplitude[c] | 1.44 (1.28-1.62) <0.001 | | | | | | |
| Acrophase[c] | | 1.01 (0.90-1.13) 0.884 | | | | | |
| Variation of cycle length[d] | | | 1.30 (1.17-1.44) <0.001 | | | | |
| IS[c] | | | | 1.28 (1.15-1.44) <0.001 | | | |
| IV[d] | | | | | 1.26 (1.12-1.42) <0.001 | | |
| M10[c] | | | | | | 1.43 (1.25-1.63) <0.001 | |
| L5[d] | | | | | | | 1.04 (0.93-1.16) 0.466 |

Cox proportional hazard regression models were used to examine the associations. Models were adjusted for age, sex, and education.
[a]Results for 1-unit increase, [b]Results for 1-unit decrease, [c]Results for 1-SD decrease, [d]Results for 1-SD increase.
Abbreviation: SD, standard deviation; HR, hazard ratio; CI, confidence interval; IS, interdaily stability; IV, intradaily variability; M10, the average activity during the most active 10-h; L5, the average activity during the least active 5-h period.
P values were from two-sided tests without correcting for multiple comparisons.

**Supplementary Table 2. Association of secondary circadian rest-activity metrics and incident frailty after contrlling for covariates**

| | 24-h amplitude | | 24-h acrophase | | Low RA | |
|---|---|---|---|---|---|---|
| | Model A HR (95%CI) *p* value | Model B HR (95%CI) *p* value | Model A HR (95%CI) *p* value | Model B HR (95%CI) *p* value | Model A HR (95%CI) *p* value | Model B HR (95%CI) *p* value |
| Age[a] | 1.09 (1.07-1.11) <0.001 | 1.09 (1.07-1.11) <0.001 | 1.09 (1.07-1.11) <0.001 | 1.09 (1.07-1.11) <0.001 | 1.09 (1.07-1.11) <0.001 | 1.09 (1.07-1.11) <0.001 |
| Female sex | 3.59 (2.54-5.08) <0.001 | 3.90 (2.70-5.64) <0.001 | 3.14 (2.22-4.42) <0.001 | 3.49 (2.43-5.01) <0.001 | 3.31 (2.35-4.67) <0.001 | 3.45 (2.40-4.95) <0.001 |
| Education[b] | 1.07 (1.02-1.11) 0.002 | 1.06 (1.02-1.11) 0.004 | 1.05 (1.01-1.09) 0.015 | 1.05 (1.01-1.09) 0.026 | 1.05 (1.01-1.09) 0.020 | 1.05 (1.01-1.09) 0.017 |
| Sleep duration[a] | | 1.04 (0.93-1.16) 0.523 | | 1.05 (0.94-1.17) 0.421 | | 1.09 (0.97-1.23) 0.133 |
| Sleep fragmentation[c] | | 1.18 (1.02-1.37) 0.029 | | 1.18 (1.02-1.36) 0.028 | | 1.18 (1.02-1.36) 0.027 |
| Vascular disease burden | | 1.13 (0.96-1.33) 0.148 | | 1.23 (1.04-1.44) 0.013 | | 1.20 (1.02-1.42) 0.026 |
| Vascular risk factors | | 1.25 (1.08-1.45) 0.003 | | 1.25 (1.07-1.45) 0.004 | | 1.23 (1.06-1.43) 0.007 |
| 24-h amplitude[d] | 1.45 (1.29-1.63) <0.001 | 1.39 (1.23-1.58) <0.001 | | | | |
| 24-h acrophase[d] | | | 1.03 (0.92-1.15) 0.634 | 1.06 (0.93-1.21) 0.370 | | |
| Low RA | | | | | 1.41 (1.13-1.76) 0.002 | 1.33 (1.03-1.71) 0.028 |

Cox proportional hazard regression models were used to examine the associations.
Model A was adjusted for age, sex, and education.
Model B was further adjusted for sleep duration, sleep fragmentation, vascular disease burden, and vascular risk factors.
[a]Results for 1-unit increase, [b]Results for 1-unit decrease, [c]Results for 1-SD increase, [d]Results for 1-SD decrease.
Abbreviation: SD, standard deviation; HR, hazard ratio; CI, confidence interval; RA, relative amplitude.
P values were from two-sided tests without correcting for multiple comparisons.

**Supplementary Table 3. Sensitivity analyses examining the association of circadian rest-activity metrics and incident frailty with adjustment for all covariates**

| | Amplitude[a] | Acrophase[a] | Variation of cycle length[b] | IS[a] | IV[b] | M10[a] | L5[b] |
|---|---|---|---|---|---|---|---|
| | HR (95%CI) *p* value | HR (95%CI) *p* value | HR (95%CI) *p* value | HR (95%CI) *p* value | HR (95%CI) *p* value | HR (95%CI) *p* value | HR (95%CI) *p* value |
| 1) Competing risk regression (n=1022) | 1.17 (1.04-1.31) 0.009 | 1.00 (0.90-1.11) 0.980 | 1.19 (1.07-1.32) 0.002 | 1.19 (1.06-1.34) 0.004 | 1.03 (0.92-1.16) 0.610 | 1.22 (1.05-1.42) 0.011 | 1.10 (0.96-1.27) 0.18 |
| 2) Controlling for AD&PD at baseline (n=1019) | 1.32 (1.17-1.50) <0.001 | 1.05 (0.93-1.17) 0.447 | 1.21 (1.07-1.36) 0.003 | 1.23 (1.09-1.38) 0.001 | 1.17 (1.03-1.33) 0.019 | 1.36 (1.16-1.58) <0.001 | 1.14 (0.98-1.32) 0.091 |
| 3) Excluding persons with baseline and incident cognitive impairment (n=473) | 1.34 (1.09-1.64) 0.006 | 1.05 (0.87-1.27) 0.624 | 1.31 (1.08-1.59) 0.007 | 1.28 (1.05-1.55) 0.014 | 1.12 (0.91-1.38) 0.282 | 1.30 (0.99-1.72) 0.063 | 1.24 (0.99-1.54) 0.056 |
| 4) In participants with lower physical activity (n=511) | 1.29 (1.07-1.54) 0.006 | 1.06 (0.88-1.27) 0.529 | 1.33 (1.13-1.58) 0.001 | 1.40 (1.18-1.66) <0.001 | 1.09 (0.92-1.31) 0.316 | 1.57 (1.31-1.88) <0.001 | 1.18 (0.93-1.49) 0.182 |
| 5) Stratifying participants by age at baseline | | | | | | | |
| <80 years old (n=416) | 1.27 (1.04-1.56) 0.018 | 1.08 (0.87-1.33) 0.478 | 1.21 (0.96-1.52) 0.106 | 1.20 (0.97-1.47) 0.091 | 1.28 (1.04-1.56) 0.018 | 1.43 (1.11-1.82) 0.005 | 1.25 (0.95-1.64) 0.115 |
| ≥80 years old (n=606) | 1.38 (1.18-1.62) <0.001 | 1.03 (0.89-1.18) 0.731 | 1.32 (1.14-1.52) <0.001 | 1.29 (1.12-1.50) <0.001 | 1.11 (0.95-1.31) 0.197 | 1.33 (1.10-1.61) 0.003 | 1.16 (0.97-1.39) 0.102 |

We performed 1) Competing risk regression models with death as a competing risk; 2) Cox proportional hazards models after further controlling for the clinical Alzheimer's disease and Parkinson's disease diagnoses at baseline; 3) Cox models by excluding participants who had cognitive impairment at baseline or developed cognitive impairment during follow-up assessments; 4) Cox models in participants with relatively lower actigraphy-derived total daily activity levels (i.e., lower than the cohort median); 5) Cox models separately for individuals under 80 years old and those aged 80 years old and above. All these models were fully adjusted for age, sex, education, sleep duration, sleep fragmentation, vascular disease burden, and vascular disease risk.

[a]Results for 1-SD decrease, [b]Results for 1-SD increase.

Abbreviation: AD, Alzheimer's disease; PD, Parkinson's disease; SD, standard deviation; HR, hazard ratio; CI, confidence interval; IS, interdaily stability; IV, intradaily variability; M10, the average activity during the most active 10-h; L5, the average activity during the least active 5-h period. P values were two-sided tests without controlling for multiple comparisons.

**Supplementary Table 4. Circadian rest-activity metrics and change in frailty with adjustment for covariates**

| | Estimate (SE) *p* value | Estimate (SE) *p* value | Estimate (SE) *p* value | Estimate (SE) *p* value | Estimate (SE) *p* value | Estimate (SE) *p* value | Estimate (SE) *p* value |
|---|---|---|---|---|---|---|---|
| Intercept | -0.118 (0.027) <0.001 | -0.125 (0.027) <0.001 | -0.123 (0.027) <0.001 | -0.122 (0.027) <0.001 | -0.121 (0.027) <0.001 | -0.114 (0.027) <0.001 | -0.127 (0.027) <0.001 |
| Time | 0.089 (0.004) <0.001 | 0.088 (0.004) <0.001 | 0.088 (0.004) <0.001 | 0.088 (0.004) <0.001 | 0.088 (0.004) <0.001 | 0.090 (0.004) <0.001 | 0.088 (0.004) <0.001 |
| Age | 0.029 (0.002) <0.001 | 0.032 (0.002) <0.001 | 0.031 (0.002) <0.001 | 0.031 (0.002) <0.001 | 0.029 (0.002) <0.001 | 0.029 (0.002) <0.001 | 0.031 (0.002) <0.001 |
| Age × time | 0.002 (0.0003) <0.001 | 0.002 (0.0003) <0.001 | 0.002 (0.0003) <0.001 | 0.002 (0.0003) <0.001 | 0.002 (0.0003) <0.001 | 0.002 (0.0003) <0.001 | 0.002 (0.0003) <0.001 |
| Sex (male) | -0.045 (0.035) 0.204 | -0.023 (0.035) 0.507 | -0.043 (0.035) 0.221 | -0.043 (0.035) 0.225 | -0.051 (0.035) 0.150 | -0.045 (0.035) 0.201 | -0.028 (0.035) 0.423 |
| Sex (male) × time | -0.007 (0.005) 0.188 | -0.007 (0.005) 0.172 | -0.005 (0.005) 0.301 | -0.007 (0.005) 0.191 | -0.007 (0.005) 0.186 | -0.007 (0.005) 0.179 | -0.006 (0.005) 0.256 |
| Education | -0.007 (0.005) 0.178 | -0.005 (0.005) 0.343 | -0.005 (0.005) 0.291 | -0.006 (0.005) 0.230 | -0.007 (0.005) 0.179 | -0.007 (0.005) 0.176 | -0.005 (0.005) 0.352 |
| Education × time | 0.0002 (0.001) 0.771 | 0.0004 (0.001) 0.574 | 0.0005 (0.001) 0.525 | 0.0004 (0.001) 0.603 | 0.0004 (0.001) 0.626 | 0.0003 (0.001) 0.674 | 0.0005 (0.001) 0.550 |
| Sleep duration | 0.017 (0.014) 0.243 | 0.027 (0.015) 0.063 | 0.024 (0.015) 0.096 | 0.020 (0.015) 0.165 | 0.012 (0.015) 0.432 | -0.001 (0.016) 0.940 | 0.030 (0.018) 0.086 |
| Sleep duration × time | 0.001 (0.002) 0.566 | 0.0004 (0.002) 0.856 | 0.001 (0.002) 0.550 | 0.001 (0.002) 0.517 | 0.001 (0.002) 0.667 | -0.001 (0.002) 0.810 | 0.002 (0.003) 0.503 |
| Sleep fragmentation[a] | 0.007 (0.020) 0.720 | 0.016 (0.020) 0.436 | 0.013 (0.020) 0.507 | 0.014 (0.020) 0.485 | 0.004 (0.020) 0.845 | 0.002 (0.020) 0.932 | 0.015 (0.020) 0.462 |
| Sleep fragmentation × time[b] | 0.002 (0.003) 0.512 | 0.001 (0.003) 0.670 | 0.002 (0.003) 0.515 | 0.002 (0.003) 0.474 | 0.002 (0.003) 0.558 | 0.001 (0.003) 0.798 | 0.002 (0.003) 0.475 |
| Vascular disease burden | 0.049 (0.025) 0.044 | 0.056 (0.024) 0.021 | 0.054 (0.024) 0.029 | 0.057 (0.024) 0.020 | 0.052 (0.024) 0.032 | 0.051 (0.025) 0.039 | 0.058 (0.025) 0.018 |
| Vascular disease burden × time | 0.004 (0.004) 0.283 | 0.005 (0.004) 0.204 | 0.005 (0.004) 0.195 | 0.005 (0.004) 0.216 | 0.005 (0.004) 0.205 | 0.004 (0.004) 0.274 | 0.005 (0.004) 0.206 |
| Vascular risk factors | -0.040 (0.018) 0.033 | -0.039 (0.019) 0.037 | -0.035 (0.019) 0.058 | -0.037 (0.019) 0.046 | -0.035 (0.018) 0.056 | -0.042 (0.019) 0.023 | -0.035 (0.019) 0.060 |

| | | | | | | | |
|---|---|---|---|---|---|---|---|
| Vascular risk factors × time | 0.005 (0.003)<br>0.072 | 0.005 (0.003)<br>0.047 | 0.005 (0.003)<br>0.079 | 0.005 (0.003)<br>0.062 | 0.005 (0.003)<br>0.060 | 0.004 (0.003)<br>0.109 | 0.005 (0.003)<br>0.072 |
| Amplitude[a] | -0.050 (0.016)<br>0.002 | | | | | | |
| Amplitude × time[b] | -0.005 (0.002)<br>0.030 | | | | | | |
| Acrophase[a] | | 0.039 (0.015)<br>0.010 | | | | | |
| Acrophase × time[b] | | -0.005 (0.002)<br>0.022 | | | | | |
| Variation of cycle length[a] | | | 0.039 (0.015)<br>0.010 | | | | |
| Variation of cycle length × time[b] | | | -0.002 (0.002)<br>0.423 | | | | |
| IS[a] | | | | -0.034 (0.015)<br>0.026 | | | |
| IS × time[b] | | | | -0.002 (0.002)<br>0.389 | | | |
| IV[a] | | | | | 0.052 (0.016)<br>0.001 | | |
| IV × time[b] | | | | | 0.003 (0.002)<br>0.255 | | |
| M10[a] | | | | | | -0.057 (0.018)<br>0.001 | |
| M10 × time[b] | | | | | | -0.005 (0.002)<br>0.026 | |
| L5[a] | | | | | | | 0.020 (0.022)<br>0.351 |
| L5 × time[b] | | | | | | | 0.001 (0.003)<br>0.861 |

Linear mixed-effects models were used to examine the associations. Models were adjusted for age, sex, education, sleep duration, sleep fragmentation, vascular disease burden, vascular risk factors, and their interactions with time.

[a]Results for 1-SD change, [b]Results for 1-SD ×1-year change.

Abbreviation: SD, standard deviation; SE, standard error; IS, interdaily stability; IV, intradaily variability; M10, the average activity during the most active 10-h; L5, the average activity during the least active 5-h period.
P values were from two-sided tests without correcting for multiple comparisons.

**Supplementary Table 5. Circadian rest-activity metrics and change in frailty after adjustment for demographics**

| | Estimate (SE) *p* value | Estimate (SE) *p* value | Estimate (SE) *p* value | Estimate (SE) *p* value | Estimate (SE) *p* value | Estimate (SE) *p* value | Estimate (SE) *p* value |
|---|---|---|---|---|---|---|---|
| Intercept | -0.142 (0.017) <0.001 | -0.147 (0.017) <0.001 | -0.142 (0.017) <0.001 | -0.141 (0.017) <0.001 | -0.140 (0.017) <0.001 | -0.145 (0.016) <0.001 | -0.146 (0.017) <0.001 |
| Time | 0.096 (0.003) <0.001 | 0.095 (0.002) <0.001 | 0.095 (0.003) <0.001 | 0.095 (0.003) <0.001 | 0.096 (0.003) <0.001 | 0.096 (0.003) <0.001 | 0.095 (0.002) <0.001 |
| Age | 0.031 (0.002) <0.001 | 0.033 (0.002) <0.001 | 0.033 (0.002) <0.001 | 0.033 (0.002) <0.001 | 0.030 (0.002) <0.001 | 0.030 (0.002) <0.001 | 0.032 (0.002) <0.001 |
| Age × time | 0.002 (0.0003) <0.001 | 0.002 (0.0003) <0.001 | 0.002 (0.0003) <0.001 | 0.002 (0.0003) <0.001 | 0.002 (0.0003) <0.001 | 0.002 (0.0003) <0.001 | 0.002 (0.0003) <0.001 |
| Sex (male) | -0.052 (0.034) 0.130 | -0.030 (0.034) 0.374 | -0.048 (0.034) 0.161 | -0.049 (0.034) 0.156 | -0.056 (0.034) 0.101 | -0.044 (0.034) 0.189 | -0.036 (0.034) 0.295 |
| Sex (male) × time | -0.007 (0.005) 0.145 | -0.007 (0.005) 0.165 | -0.006 (0.005) 0.268 | -0.007 (0.005) 0.172 | -0.007 (0.005) 0.160 | -0.007 (0.005) 0.152 | -0.006 (0.005) 0.211 |
| Education | -0.006 (0.005) 0.264 | -0.003 (0.005) 0.520 | -0.004 (0.005) 0.446 | -0.005 (0.005) 0.347 | -0.006 (0.005) 0.242 | -0.006 (0.005) 0.220 | -0.004 (0.005) 0.375 |
| Education × time | 0.0001 (0.001) 0.933 | 0.00002 (0.001) 0.788 | 0.0003 (0.001) 0.709 | 0.0002 (0.001) 0.787 | 0.0001 (0.001) 0.850 | 0.0004 (0.001) 0.638 | 0.0004 (0.001) 0.554 |
| Amplitude[a] | -0.058 (0.015) <0.001 | | | | | | |
| Amplitude × time[b] | -0.004 (0.002) 0.047 | | | | | | |
| Acrophase[a] | | 0.033 (0.015) 0.026 | | | | | |
| Acrophase × time[b] | | -0.004 (0.002) 0.077 | | | | | |
| Variation of cycle length[a] | | | 0.040 (0.015) 0.007 | | | | |
| Variation of cycle length × time[b] | | | -0.002 (0.002) 0.301 | | | | |
| IS[a] | | | | -0.038 (0.015) 0.011 | | | |
| IS × time[b] | | | | -0.001 (0.002) 0.603 | | | |

| | | | | | | | | | |
|---|---|---|---|---|---|---|---|---|---|
| IV[a] | 0.061 | (0.015) | <0.001 | | | | | | |
| IV × time[b] | 0.002 | (0.002) | 0.290 | | | | | | |
| M10[a] | | | | -0.062 | (0.015) | <0.001 | | | |
| M10 × time[b] | | | | -0.005 | (0.002) | 0.011 | | | |
| L5[a] | | | | | | | -0.010 | (0.014) | 0.486 |
| L5 × time[b] | | | | | | | -0.002 | (0.002) | 0.258 |

Linear mixed-effects models were used to examine the associations. Models were adjusted for age, sex, and education.

[a]Results for 1-SD change, [b]Results for 1-SD ×1-year change.

Abbreviation: SD, standard deviation; SE, standard error; IS, interdaily stability; IV, intradaily variability; M10, the average activity during the most active 10-h; L5, the average activity during the least active 5-h period.

P values were from two-sided tests without correcting for multiple comparisons.

**Supplementary Table 6. Circadian rest-activity metrics and change in frailty components after adjustment for demographics**

| | Grip strength | | Gait speed | | BMI | | Fatigue | |
|---|---|---|---|---|---|---|---|---|
| | Model A | Model B | Model A | Model B | Model A | Model B | Model A | Model B |
| | Estimate (SE) *p* value | Estimate (SE) *p* value | Estimate (SE) *p* value | Estimate (SE) *p* value | Estimate (SE) *p* value | Estimate (SE) *p* value | Estimate (SE) *p* value | Estimate (SE) *p* value |
| Amplitude[a] | 2.051 (0.389) <0.001 | 1.910 (0.408) <0.001 | -0.425 (0.054) <0.001 | -0.369 (0.051) <0.001 | -0.966 (0.170) <0.001 | -1.000 (0.177) <0.001 | -0.258 (0.074) <0.001 | -0.244 (0.078) 0.002 |
| Amplitude × time[b] | 0.069 (0.046) 0.137 | 0.078 (0.048) 0.104 | -0.026 (0.014) 0.059 | -0.023 (0.014) 0.112 | 0.053 (0.015) <0.001 | 0.056 (0.015) <0.001 | 0.001 (0.009) 0.918 | 0.001 (0.009) 0.929 |
| Acrophase[a] | -0.494 (0.383) 0.197 | -0.136 (0.395) 0.732 | 0.034 (0.054) 0.526 | 0.087 (0.050) 0.081 | -0.160 (0.169) 0.344 | -0.217 (0.172) 0.207 | 0.248 (0.073) 0.001 | 0.196 (0.077) 0.010 |
| Acrophase × time[b] | 0.044 (0.047) 0.351 | 0.045 (0.049) 0.362 | 0.003 (0.014) 0.852 | -0.004 (0.015) 0.777 | 0.028 (0.016) 0.075 | 0.032 (0.016) 0.041 | -0.020 (0.010) 0.035 | -0.015 (0.010) 0.134 |
| Variation of cycle length[a] | -1.061 (0.377) 0.005 | -0.757 (0.394) 0.055 | 0.140 (0.054) 0.009 | 0.179 (0.049) <0.001 | 0.348 (0.167) 0.038 | 0.435 (0.169) 0.010 | 0.154 (0.071) 0.029 | 0.139 (0.074) 0.061 |
| Variation of cycle length × time[b] | -0.060 (0.046) 0.193 | -0.094 (0.048) 0.052 | -0.002 (0.014) 0.911 | -0.009 (0.014) 0.541 | -0.021 (0.015) 0.155 | -0.027 (0.015) 0.062 | -0.007 (0.008) 0.409 | -0.005 (0.009) 0.600 |
| IS[a] | 0.584 (0.380) 0.124 | 0.318 (0.390) 0.414 | -0.297 (0.054) <0.001 | -0.259 (0.050) <0.001 | -0.854 (0.166) <0.001 | -0.873 (0.168) <0.001 | -0.302 (0.072) <0.001 | -0.305 (0.074) <0.001 |
| IS × time[b] | 0.119 (0.045) 0.008 | 0.131 (0.046) 0.005 | 0.003 (0.014) 0.850 | 0.002 (0.014) 0.880 | 0.037 (0.015) 0.012 | 0.039 (0.014) 0.007 | 0.007 (0.009) 0.451 | 0.006 (0.009) 0.495 |
| IV[a] | -1.635 (0.394) <0.001 | -1.634 (0.413) <0.001 | 0.413 (0.055) <0.001 | 0.292 (0.052) <0.001 | 0.584 (0.174) 0.001 | 0.603 (0.181) 0.001 | 0.046 (0.077) 0.547 | 0.053 (0.081) 0.513 |
| IV × time[b] | -0.142 (0.049) 0.004 | -0.141 (0.050) 0.005 | 0.015 (0.014) 0.302 | 0.019 (0.015) 0.195 | -0.026 (0.016) 0.099 | -0.030 (0.016) 0.059 | 0.007 (0.010) 0.497 | 0.004 (0.010) 0.700 |
| M10[a] | 1.126 (0.388) 0.004 | 1.437 (0.459) 0.002 | -0.575 (0.051) <0.001 | -0.447 (0.056) <0.001 | -1.028 (0.168) <0.001 | -1.190 (0.198) <0.001 | -0.193 (0.074) 0.010 | -0.294 (0.088) 0.001 |
| M10 × time[b] | 0.107 (0.045) 0.019 | 0.085 (0.051) 0.097 | -0.017 (0.013) 0.206 | -0.030 (0.015) 0.052 | 0.043 (0.015) 0.004 | 0.052 (0.016) 0.002 | -0.021 (0.009) 0.028 | -0.009 (0.010) 0.368 |
| L5[a] | -0.670 (0.371) 0.072 | -0.740 (0.509) 0.146 | -0.196 (0.052) <0.001 | 0.148 (0.071) 0.037 | 0.136 (0.164) 0.408 | 0.399 (0.223) 0.073 | 0.182 (0.068) 0.007 | 0.192 (0.093) 0.039 |
| L5 × time[b] | 0.066 (0.046) 0.151 | -0.037 (0.068) 0.591 | 0.014 (0.013) 0.270 | 0.003 (0.019) 0.862 | -0.002 (0.016) 0.891 | -0.009 (0.021) 0.688 | -0.019 (0.008) 0.023 | -0.015 (0.011) 0.180 |

Linear mixed-effects models were used to examine the associations.

Model A was adjusted for age, sex, and education.
Model B was further adjusted for sleep duration, sleep fragmentation, vascular disease burden, and vascular risk factors.
[a]Results for 1-SD change, [b]Results for 1-SD ×1-year change.
Abbreviation: BMI, body mass index; SD, standard deviation; SE, standard error; IS, interdaily stability; IV, intradaily variability; M10, the average activity during the most active 10-h; L5, the average activity during the least active 5-h period.
P values were from two-sided tests without correcting for multiple comparisons.

**Supplementary Table 7. STROBE checklist**

| | Item No. | Recommendation | Page No. | Relevant text from manuscript |
|---|---|---|---|---|
| **Title and abstract** | 1 | (*a*) Indicate the study's design with a commonly used term in the title or the abstract | Page 1 | Abstract |
| | | (*b*) Provide in the abstract an informative and balanced summary of what was done and what was found | Page 1 | Abstract |
| **Introduction** | | | | |
| Background/rationale | 2 | Explain the scientific background and rationale for the investigation being reported | Page 2 | Introduction Paragraph1,2 |
| Objectives | 3 | State specific objectives, including any prespecified hypotheses | Page 2 | Introduction Paragraph3 |
| **Methods** | | | | |
| Study design | 4 | Present key elements of study design early in the paper | Page 6-7 | Study design |
| Setting | 5 | Describe the setting, locations, and relevant dates, including periods of recruitment, exposure, follow-up, and data collection | Page 6-7 | Study design |
| Participants | 6 | (*a*) *Cohort study*—Give the eligibility criteria, and the sources and methods of selection of participants. Describe methods of follow-up | Page 7 | Participants |
| | | (*b*) *Cohort study*—For matched studies, give matching criteria and number of exposed and unexposed | Not applicable | Not applicable |
| Variables | 7 | Clearly define all outcomes, exposures, predictors, potential confounders, and effect modifiers. Give diagnostic criteria, if applicable | Page 7-9 | Assessment of circadian rest-activity rhythms, Assessment of frailty, Covariates |
| Data sources/ measurement | 8* | For each variable of interest, give sources of data and details of methods of assessment (measurement). Describe comparability of assessment methods if there is more than one group | Page 7-9 | Assessment of circadian rest-activity rhythms, Assessment of frailty, Covariates |
| Bias | 9 | Describe any efforts to address potential sources of bias | Page 8-9 | Covariates |
| Study size | 10 | Explain how the study size was arrived at | Page 7 | Participants |
| Quantitative variables | 11 | Explain how quantitative variables were handled in the analyses. If applicable, describe which groupings were chosen and why | Not applicable | Not applicable |
| Statistical methods | 12 | (*a*) Describe all statistical methods, including those used to control for confounding | Page 9 | Statistical analysis |
| | | (*b*) Describe any methods used to examine subgroups and interactions | Page 9 | Statistical analysis |
| | | (*c*) Explain how missing data were addressed | Page 7 | Participants |

| | | | | |
|---|---|---|---|---|
| | | (*d*) *Cohort study*—If applicable, explain how loss to follow-up was addressed | Page 7 | Participants |
| | | (*e*) Describe any sensitivity analyses | Page 9 | Statistical analysis |
| **Results** | | | | |
| Participants | 13* | (a) Report numbers of individuals at each stage of study—eg numbers potentially eligible, examined for eligibility, confirmed eligible, included in the study, completing follow-up, and analysed | Page 2 | Results Paragraph1 |
| | | (b) Give reasons for non-participation at each stage | Page 2 | Results Paragraph1 |
| | | (c) Consider use of a flow diagram | Page 8 | Fig. 3 |
| Descriptive data | 14* | (a) Give characteristics of study participants (eg demographic, clinical, social) and information on exposures and potential confounders | Page 2, 17 | Results Paragraph1, Table 1 |
| | | (b) Indicate number of participants with missing data for each variable of interest | Not applicable | Not applicable |
| | | (c) *Cohort study*—Summarise follow-up time (eg, average and total amount) | Page 3, 7 | Results Paragraph2, Participants |
| Outcome data | 15* | *Cohort study*—Report numbers of outcome events or summary measures over time | Page 2 | Results Paragraph2 |
| Main results | 16 | (*a*) Give unadjusted estimates and, if applicable, confounder-adjusted estimates and their precision (eg, 95% confidence interval). Make clear which confounders were adjusted for and why they were included | Page 17-20, Supplementary file | Table 2-3, Supplementary Table 1-6 |
| | | (*b*) Report category boundaries when continuous variables were categorized | Not applicable | Not applicable |
| | | (*c*) If relevant, consider translating estimates of relative risk into absolute risk for a meaningful time period | Not applicable | Not applicable |
| Other analyses | 17 | Report other analyses done—eg analyses of subgroups and interactions, and sensitivity analyses | Page 3, Supplementary file | Results Paragraph3, Supplementary Table 3 |
| **Discussion** | | | | |
| Key results | 18 | Summarise key results with reference to study objectives | Page 4 | Discussion Paragraph1 |
| Limitations | 19 | Discuss limitations of the study, taking into account sources of potential bias or imprecision. Discuss both direction and magnitude of any potential bias | Page 6 | Discussion Paragraph8 |
| Interpretation | 20 | Give a cautious overall interpretation of results considering objectives, limitations, multiplicity of analyses, results from similar studies, and other relevant evidence | Page 4-5 | Discussion Paragraph2,5 |
| Generalisability | 21 | Discuss the generalisability (external validity) of the study results | Page 6 | Discussion Paragraph9 |
| Other information | | | | |
| **Funding** | 22 | Give the source of funding and the role of the funders for the present study and, if applicable, for the original study on which the present article is based | Page 15 | Acknowledgements |

*Give information separately for cases and controls in case-control studies and, if applicable, for exposed and unexposed groups in cohort and cross-sectional studies.

**Note:** An Explanation and Elaboration article discusses each checklist item and gives methodological background and published examples of transparent reporting. The STROBE checklist is best used in conjunction with this article (freely available on the Web sites of PLoS Medicine at http://www.plosmedicine.org/, Annals of Internal Medicine at http://www.annals.org/, and Epidemiology at http://www.epidem.com/). Information on the STROBE Initiative is available at www.strobe-statement.org.
