# Supplementary material for: Circadian disturbances and frailty risk in older adults
Source: Nat Commun. 2023 Nov 16;14:7219. doi: 10.1038/s41467-023-42727-z (PMC10654720; doi:10.1038/s41467-023-42727-z)
Supplement: Supplementary file 2 — Reporting Summary [file 41467_2023_42727_MOESM2_ESM.pdf]

nature portfolio

Corresponding author(s): Peng Li
Last updated by author(s): Oct 5, 2023

Reporting Summary

Nature Portfolio wishes to improve the reproducibility of the work that we publish. This form provides structure for consistency and transparency in reporting. For further information on Nature Portfolio policies, see our Editorial Policies and the Editorial Policy Checklist.

Statistics

For all statistical analyses, confirm that the following items are present in the figure legend, table legend, main text, or Methods section.

n/a	Confirmed	
☐	☒	The exact sample size (*n*) for each experimental group/condition, given as a discrete number and unit of measurement
☐	☒	A statement on whether measurements were taken from distinct samples or whether the same sample was measured repeatedly
☐	☒	The statistical test(s) used AND whether they are one- or two-sided<br>*Only common tests should be described solely by name; describe more complex techniques in the Methods section.*
☐	☒	A description of all covariates tested
☐	☒	A description of any assumptions or corrections, such as tests of normality and adjustment for multiple comparisons
☐	☒	A full description of the statistical parameters including central tendency (e.g. means) or other basic estimates (e.g. regression coefficient) AND variation (e.g. standard deviation) or associated estimates of uncertainty (e.g. confidence intervals)
☐	☒	For null hypothesis testing, the test statistic (e.g. *F*, *t*, *r*) with confidence intervals, effect sizes, degrees of freedom and *P* value noted<br>*Give P values as exact values whenever suitable.*
☒	☐	For Bayesian analysis, information on the choice of priors and Markov chain Monte Carlo settings
☒	☐	For hierarchical and complex designs, identification of the appropriate level for tests and full reporting of outcomes
☐	☒	Estimates of effect sizes (e.g. Cohen's *d*, Pearson's *r*), indicating how they were calculated

Our web collection on statistics for biologists contains articles on many of the points above.

Software and code

Policy information about availability of computer code

Data collection	No software was used for data collection.
Data analysis	A customized software App, "ezActi2", developed in MATLAB (version R2022a) was used for the rest-activity rhythms analysis in this manuscript. The App is available at Zenodo (https://doi.org/10.5281/zenodo.8411607). Statistical analyses were performed in R (version 4.1.2) using the following packages: survival, lme4, lmerTest, cmprsk, ggplot2. No custom code was used in the statistical analyses.

For manuscripts utilizing custom algorithms or software that are central to the research but not yet described in published literature, software must be made available to editors and reviewers. We strongly encourage code deposition in a community repository (e.g. GitHub). See the Nature Portfolio guidelines for submitting code & software for further information.

Data

Policy information about availability of data

All manuscripts must include a data availability statement. This statement should provide the following information, where applicable:

- Accession codes, unique identifiers, or web links for publicly available datasets
- A description of any restrictions on data availability
- For clinical datasets or third party data, please ensure that the statement adheres to our policy

The data are available under restricted access for the Rush Alzheimer's Disease Center (RADC) data and resource sharing policy, access can be obtained by submitting requests through the RADC platform https://www.radc.rush.edu/requests.htm. Source data supporting our findings (Fig. 1, Fig. 2, and Supplementary Fig. 1) are provided with this paper.

Human research participants

Policy information about studies involving human research participants and Sex and Gender in Research.

Reporting on sex and gender	762 (74.6%)were female, 260 (25.4%) were male.
Population characteristics	The mean age at baseline was 81 years old (standard deviation: 7.2; range: 59-100 years old). Among them, 74.6% were female.
Recruitment	We studied participants in the Rush Memory and Aging Project (MAP), an ongoing prospective, observational cohort study conducted at the Rush Alzheimer's Disease Center, Rush University Medical Center. The MAP started in 1997, and over 2,100 participants have agreed to receive annual clinical evaluations, cognitive and frailty assessments. Starting in 2005, actigraphy (i.e., collection of daily motor activity data with an omnidirectional accelerometer) was added to the annual visit in the MAP. In the current study, the visit when the first actigraphy assessment was performed was used as the analytic baseline for each participant. While all alive MAP participants enrolled prior to 2005 and new enrolled participants are eligible for the actigraphy sub-study, not everyone agreed to wear it. Compared to those who did not participate in the actigraphy assessment, participants who completed the actigraphy assessment were younger and less frail, and had higher levels of education, but they did not differ with respect to sex, vascular disease burden, and vascular risk factors.
Ethics oversight	The MAP was approved by an Institutional Review Board of Rush University Medical Center. All participants signed an informed consent and a repository consent to allow their data to be shared with broader research community. The current study was reviewed and approved by the Institutional Review Board of Mass General Brigham.

Note that full information on the approval of the study protocol must also be provided in the manuscript.

Field-specific reporting

Please select the one below that is the best fit for your research. If you are not sure, read the appropriate sections before making your selection.

☒ Life sciences ☐ Behavioural & social sciences ☐ Ecological, evolutionary & environmental sciences

For a reference copy of the document with all sections, see nature.com/documents/nr-reporting-summary-flat.pdf

Life sciences study design

All studies must disclose on these points even when the disclosure is negative.

Sample size	The sample size in this study is 1,022. The sample size was determined base on the available participants in this database that met inclusion and exclusion criteria. This is a cohort study assessing exposure-outcome associations. For time-to-event outcomes, a well-known rule of thumb for the required sample size is to ensure at least 10 events for each predictor parameter. We included a total of 17 variables in the fully adjusted Cox regression models. Thus, 170 events were required, whereas the frailty events in this study were 357. Therefore, the sample size of this study should be sufficient.
Data exclusions	A total of 1,401 participants completed baseline actigraphy assessment. Among them, 218 participants were frail or did not have frailty assessment at baseline actigraphy assessment, and they were excluded. After that, 123 participants who did not have a follow-up frailty assessment and 38 individuals who died prior to be followed were further excluded. This resulted in 1,022 participants who entered the subsequent analyses.
Replication	This is a population-based cohort study, and we have not yet replicated the findings in other datasets. In order to ensure the reproducibility of the study, various statistical analyses were performed: 1) Cox proportional hazards models controlling for a wide range of potential covariates, 2) comprehensive sensitivity analyses [performed competing risk regression models with death as a competing risk; repeated the Cox proportional hazards models after further controlling for the clinical Alzheimer's disease and Parkinson's disease diagnoses at baseline; repeated the Cox models by excluding participants who had cognitive impairment at baseline or developed cognitive impairment during follow-up assessments; restricted the Cox models to participants with relatively lower actigraphy-derived total daily activity levels; stratified

	participants by age and repeated the Cox models separately for individuals under 80 years old and those aged 80 years old and above], 3) linear mixed-effects models evaluating the longitudinal change. Our findings were consistent across these analyses.
Randomization	N/A as it is an observational study.
Blinding	N/A as it is an observational study.

Reporting for specific materials, systems and methods

We require information from authors about some types of materials, experimental systems and methods used in many studies. Here, indicate whether each material, system or method listed is relevant to your study. If you are not sure if a list item applies to your research, read the appropriate section before selecting a response.

Materials & experimental systems

n/a	Involved in the study
☒	☐ Antibodies
☒	☐ Eukaryotic cell lines
☒	☐ Palaeontology and archaeology
☒	☐ Animals and other organisms
☒	☐ Clinical data
☒	☐ Dual use research of concern

Methods

n/a	Involved in the study
☒	☐ ChIP-seq
☒	☐ Flow cytometry
☒	☐ MRI-based neuroimaging
